# Transcriptome Analysis Revealed That Cell Wall Regulatory Pathways Are Involved in the Tolerance of *Pleurotus ostreatus* Mycelia to Different Heat Stresses

**DOI:** 10.3390/jof11040266

**Published:** 2025-03-30

**Authors:** Ludan Hou, Jingyi Wang, Tonglou Li, Baosheng Zhang, Kexing Yan, Zehua Zhang, Xueran Geng, Mingchang Chang, Junlong Meng

**Affiliations:** 1 College of Food Science and Engineering, Shanxi Agricultural University, Taigu, Jinzhong 030801, China; wangjingyi0304@163.com (J.W.); 15582690226@163.com (T.L.); 15291884191@163.com (B.Z.); yan15934475351@163.com (K.Y.); zehuazhang@sxau.edu.cn (Z.Z.); gengxueran2007@163.com (X.G.); sxndcmc@163.com (M.C.); 2 Shanxi Key Laboratory of Edible Fungi for Loess Plateau, Taigu, Jinzhong 030801, China; 3 Shanxi Research Center for Engineering Technology of Edible Fungi, Taigu, Jinzhong 030801, China

**Keywords:** *Pleurotus ostreatus*, heat stress, RNA-seq, cell wall

## Abstract

*Pleurotus ostreatus* is the third largest cultivated species in China’s edible mushroom industry; however, its agricultural cultivation method is easily affected by high-temperature environments. To understand the response mechanism of mycelia to heat stress, the mycelia of *P. ostreatus*, which had been grown at 28 °C for 4 days, were subjected to heat stress at 32 °C and 36 °C for 2 days, followed by RNA-seq analysis. These results indicate that, under heat stress, mycelial growth was significantly inhibited, the cell membrane was disrupted, the cell walls became thicker, and chitinase and β-1,3-glucanase activities decreased. Transcriptome analysis revealed 2118 differentially expressed genes (DEGs) under 36 °C heat stress, and 458 DEGs were identified under 32 °C heat stress. A total of 328 DEGs were upregulated or downregulated under heat stress at 36 °C and 32 °C. The functional enrichment analysis of these genes revealed significant enrichment in genes related to hydrogen peroxide metabolism, oxidoreductase activity, ATP hydrolysis, and cell wall structure composition. There was a total of 80 DEGs specific to heat stress at 32 °C, and they were significantly enriched in catalase activity, the cell wall, the aminoglycan catabolic process, and oxidoreductase activity. However, 817 DEGs specific to heat stress at 36 °C were significantly enriched in the cell wall, integral components of the membrane, and oxidoreductase activity. The identification of cell wall-related genes revealed that hydrophobic proteins, Cerato plateau proteins, laccases, and glycoside hydrolases may respond to stress. The results of qRT-PCR for cell wall-related genes are consistent with the RNA-seq data. This study revealed several potential candidate genes for high-temperature thermal response, laying the foundation for the study of the thermal response mechanism of *P. ostreatus*.

## 1. Introduction

*Pleurotus ostreatus* has advantages such as wide adaptability, strong viability, a short growth cycle, high yield, and fast return to investment, and it is a globally distributed edible mushroom [1]. The growth and development process of *P. ostreatus* is influenced by various environmental conditions, such as temperature, air humidity, light, and air. Among these factors, high temperature is the most susceptible abiotic stress to *P. ostreatus* production [2]. High temperature not only causes physiological and metabolic disorders in the mycelia of *P. ostreatus* but also affects the growth and development of *P. ostreatus*, including bursts of reactive oxygen species (ROS), membrane peroxidation, and cell apoptosis. High-temperature stress can damage the cell wall structure of mycelia, reduce the mannose content, and increase the relative porosity [3]. Heat stress in *P. ostreatus* can promote the degradation of fungal nucleotides and unsaturated fatty acids, increase the contents of vitamins and amino acids, and accelerate glycolysis and the tricarboxylic acid cycle [4]. Previous studies have shown that the metacaspase gene (*PoMCA1*), methionine sulfoxide reductase gene (MsrA), and phenylalanine ammonia lyase (PAL) genes affect the heat resistance of *P. ostreatus* mycelia [5,6,7]. Treatment with NO and salicylic acid can increase the heat resistance of *P. ostreatus* mycelia [2,8]. However, the mechanism of the heat stress response in the mycelia of *P. ostreatus* is still unclear, and there is a lack of candidate gene libraries for studying the mechanism of the heat stress response.

The cell wall is a thick, tough, and slightly elastic structure located outside the cell membrane that plays a role in maintaining cell shape and protecting the internal structure of the cell [9]. The cell wall is an important feature in plants, fungi, bacteria, and certain protists. The composition and structure of cell walls vary depending on the type of organism. In plants, cell walls can respond to various abiotic stresses, such as drought, floods, heat, cold, salt, heavy metals, and light and air pollutants [10]. For example, in *Picea mariana* seedlings, drought stress induces a decrease in cell wall thickness [11]. In rice seedlings and strawberry plants, high temperature can lead to differential protein expression in the lignin biosynthesis pathway, thereby affecting the inhibition of lignification [12,13]. In pea, *Allium fistulosum*, and *Arabidopsis thaliana*, changes in pectin and hemicellulose contents during cold acclimation are assumed to lead to a finer cell wall mesh and cell wall strengthening, contributing to acquired freezing tolerance [14,15,16,17]. In fungi, there have also been reports clarifying the importance of cell walls under abiotic stress. Compared with that under glucose growth conditions, the elasticity of the cell wall of *Candida albicans* under lactic acid growth conditions is lower, thereby enhancing its resistance to high osmotic environments [18]. The small cell wall protein PGA26 is necessary for the virulence of *C. albicans*, and the absence of *PGA26* may lead to an imbalance in the ability of Candida to undergo morphological transformation, resulting in reduced dissemination and infection [19]. The activity of cell wall-associated glyceraldehyde-3-phosphate dehydrogenase (cwGAPDH) in *Saccharomyces cerevisiae* increases due to starvation and increased temperature [20]. However, relatively few studies have been conducted on edible mushrooms.

*P. ostreatus* is a typical heterologous edible fungus with a short cultivation period and the potential to become a model organism. This study focused on the CCMSSC00389 strains of *P. ostreatus*. The growth inhibition rates of *P. ostreatus* mycelia under heat stress at 32 °C and 36 °C were measured, and changes in the cell wall morphology of *P. ostreatus* mycelia under heat stress were observed via transmission electron microscopy. Further transcriptome analysis revealed that genes related to oxidoreductase activity, hydrogen peroxide metabolism, ATP hydrolysis, and cell wall composition play important roles in the response of *P. ostreatus* mycelia to heat stress. This study lays the foundation for the screening of candidate genes involved in the thermal response of edible mushrooms.

## 2. Materials and Methods

### 2.1. Strains and Culture Conditions

The *P. ostreatus* CCMSSC 00389 strain, which is currently stored in the germplasm resource library of Shanxi Agricultural University, was used in this study. The genome of the CCMSSC 00389 strain was obtained from DDBJ/EMBL/GenBank under registration number MAYC 000000 [21]. The strains were inoculated onto potato glucose agar (PDA) plates, incubated in the dark at 28 °C for 4 days, and then incubated at 28 °C, 32 °C, and 36 °C for 2 days. Photos were taken, data were recorded for each stage, and mycelial samples were collected for storage in liquid nitrogen.

### 2.2. Electron Microscopy Observation and Physiological Index Determination

After heat stress, the mycelia were fixed, rinsed, dehydrated, embedded, and sliced for later use [22]. The ultrastructures of mycelia grown under heat stress at different temperatures were observed via transmission electron microscopy (TEM) with a JEM-2100F instrument (JEOL, Tokyo, Japan). Enzyme activity was detected via a chitinase and β-1,3-glucanase activity detection kit (TransGen Biotech, Beijing, China). SPSS 26 software was used for statistical analysis. GraphPad Prism 11 and Photoshop 2023 software were used for figure analysis.

### 2.3. RNA Sequencing (RNA-Seq)

Mycelia cultured at 28 °C for 6 days were used as the control group (M28), and after being cultured at 28 °C for 4 days, the mycelia were subjected to stress treatment at 32 °C and 36 °C for 2 days as the experimental group (M32, M36). Each group had 3 replicates, totaling 9 samples. Majorbio (Shanghai, China) was used to conduct transcriptome sequencing analysis, total RNA was extracted via TRIzol, and an RNA-Seq library was constructed using the TruSeqTM RNA Sample Preparation Kit (Illumina, San Diego, CA, USA). The Illumina NovaSeq Reagent Kit method was used to construct the library, followed by quality evaluation of the raw data. Splicing mapped reads on the basis of the reference genome sequence of the CCMSSC00389 strain.

### 2.4. Differential Expression Analysis and Functional Annotation

According to the statistical significance analysis, in paired comparisons (M28&M32 or M28&M36), *p*-adjust < 0.05 and multiple differences ≥ 2 were used to identify DEGs. Gene sets (up/downM28&M32, up/downM28&M36) were created on the basis of the expression levels of genes in different sample groups, and common and unique genes between gene sets were identified. The software Goatools 1.4.12 was used to perform GO enrichment analysis on the genes in the gene set to obtain the main GO functions of the genes in the gene set [23]. An R script was used to perform KEGG pathway enrichment analysis on genes in the gene set. Cluster analysis and the correlation analysis of genes are based on their expression patterns. A BLAST search of the NCBI database (https://blast.ncbi.nlm.nih.gov/Blast.cgi) (accessed on 15 October 2024) was performed to predict protein function.

### 2.5. Quantitative Real-Time PCR

A quantitative real-time PCR (qRT–PCR) assay was performed to validate the transcriptome sequencing results. After heat stress treatment, the mycelia were collected and immediately frozen in liquid nitrogen. The total RNA of each sample was extracted using the E.Z.N.A. Plant RNA Kit (Omega, Norcross, GA, USA), and the RNA was converted to cDNA via a HiScript II 1st Strand cDNA Synthesis Kit (Vazyme Biotech, Nanjing, China). Gene expression was determined via qRT-PCR with TransStart^®^ Tip Green qPCR SuperMix (+Dye II) (TransGen Biotech, Beijing, China). *β-tubulin* was used as an internal reference gene, and each data point was repeated three times [24]. The relative gene expression was analysed according to the 2^−∆∆CT^ method. The primers used in this study for qRT-PCR are shown in Supplementary Table S1.

## 3. Results

### 3.1. Evaluation and Electron Microscopy Observation of the Heat Resistance of P. ostreatus mycelia

**Figure 1:**
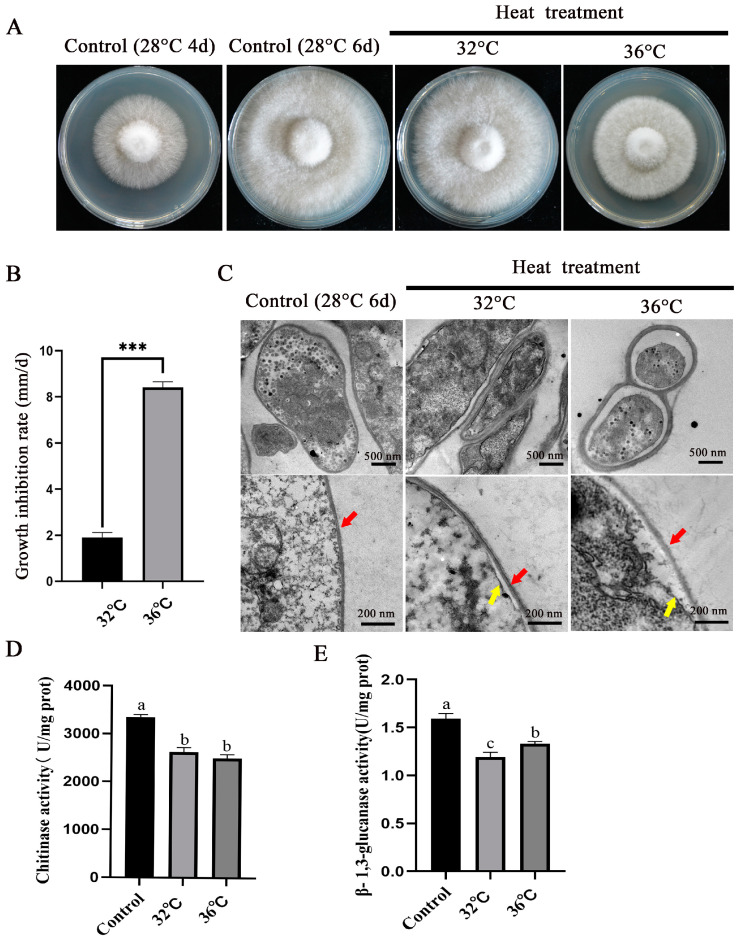
Effects of different temperature stresses on the mycelia of *P. ostreatus*: (**A**) Colony morphology of mycelia grown at 28 °C for 4 d, followed by growth at 28 °C, 32 °C, and 36 °C for 2 d. (**B**) Growth inhibition rate of mycelia at 32 °C and 36 °C; *** represents a significant difference in the growth inhibition rate between the two (*t* test, *p* < 0.01). (**C**) TEM was used to observe the effects of different heat stress treatments on mycelia. The red arrow points to the cell wall, and the yellow arrow points to the cell membrane. (**D**,**E**) Activity determination of chitinase and β-1,3-glucanase after heat stress in mycelia. Different letters indicate significant differences for the comparison of samples (*p* < 0.05 according to Duncan’s test).

To investigate the effects of different degrees of heat stress on the mycelia of *P. ostreatus*, mycelia cultured at 28 °C for 4 days were subjected to heat stress at 32 °C and 36 °C for 2 days to observe colony morphology. Figure 1A shows that, compared with mycelia grown normally at 28 °C, the colony diameter of mycelia grown at 32 °C and 36 °C was significantly reduced. Moreover, the growth inhibition rates of mycelia under heat stress at 32 °C and 36 °C were 19.07% and 84.13%, respectively (Figure 1B). These findings indicate that heat stress can significantly reduce the growth rate of mycelia. Transmission electron microscopy images (Figure 1C) revealed that the cell wall of mycelia became thicker under heat stress. The fungal cell wall is mainly composed of glucan, chitin, mannan, and glycoproteins. Further determination of the activities of chitinase and β-1,3-glucanase after heat stress (Figure 1D,E) revealed a significant decrease in the activity of both enzymes, which may lead to changes in the structural components of the cell wall.

### 3.2. RNA-Seq Data Analysis

On the basis of the phenotype of *P. ostreatus* mycelia under heat stress, mycelia subjected to heat stress were selected for further research. The transcriptome gene expression of mycelia grown at different temperatures was systematically analysed via high-throughput sequencing technology. Approximately 45.4 million, 52 million, and 57 million raw reads were obtained from groups M28, M32, and M36, respectively (Table 1). After the read quality and removal contamination were checked, the vast majority (99.2%) of high-quality clean reads (45 million, 51.6 million, and 56.7 million for the M28, M32, and M36 groups, respectively) were used for assembly and further downstream analysis. The average number of reads mapped to the reference genome was 79.58%. The percentage of unique mapped reads ranged from 78.45% to 80.08%, and these reads were available for further analyses. The detailed information about RNA sequencing and mapping is summarised in Table 1.

### 3.3. Identification of Differentially Expressed Genes (DEGs)

**Figure 2:**
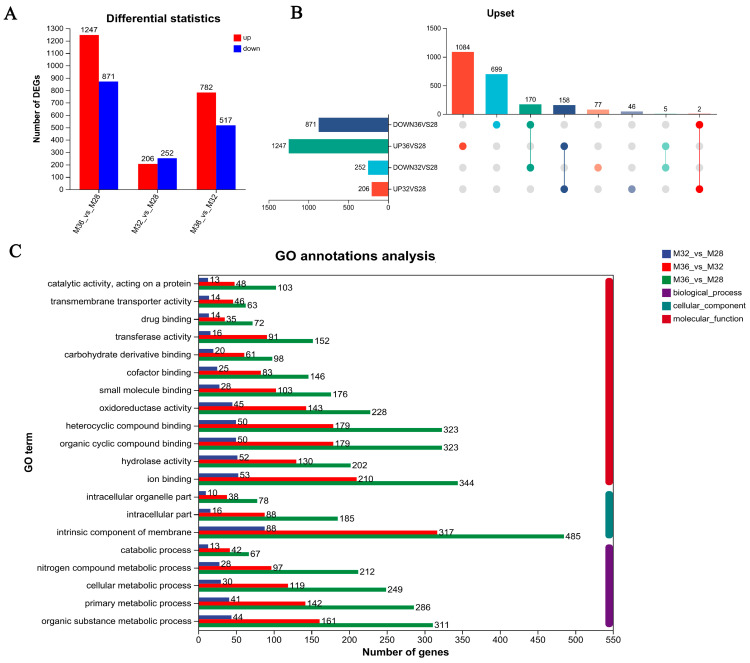
The number of upregulated and downregulated DEGs in the M28, M32, and M36 groups: (**A**) DEGs statistical chart. (**B**) Upset diagram. (**C**) GO annotation analysis.

To screen for genes related to heat stress, intergroup differential gene analysis was performed on M28, M32, and M36. Compared with those in mycelia growing at 28 °C (M28), 2118 DEGs (1247 upregulated and 871 downregulated) were detected in the mycelia growing at 36 °C, 458 DEGs (206 upregulated and 252 downregulated) were detected in the mycelia growing at 32 °C, and 1299 DEGs were detected between the M36 and M32 groups (782 upregulated and 517 downregulated) (Figure 2A). The number of DEGs produced by mycelia under 36 °C heat stress was 4.62-fold greater than that produced by mycelia under 32 °C heat stress, indicating that the response of mycelia under 36 °C heat stress was greater. There were 1783 genes with specific differential expression under 36 °C heat stress and 123 genes with specific differential expression under 32 °C heat stress. Moreover, an intergroup analysis of genes upregulated and downregulated under heat stress revealed that 158 DEGs were upregulated under 32 °C and 36 °C heat stress and that 170 DEGs were downregulated under 32 °C and 36 °C heat stress (Figure 2B). To explore the biological functions of these differentially expressed genes, GO analysis was performed on the basis of GO annotation terms. The DEGs were annotated for GO functions, and Figure 2C shows the top 20 GO terms, with more DEGs for terms such as hydrolase activity, oxidoreductase activity, membrane intrinsic components, and cellular metabolic processes. In addition, the number of DEGs was significantly greater under heat stress conditions at 32 °C and 36 °C than at 28 °C, and the number of DEGs at 36 °C was significantly greater than that at 32 °C. These findings suggest that as the degree of heat stress increased, the expression of relevant heat stress response genes increased.

### 3.4. Functional Enrichment of DEGs in Mycelia Under Different Temperature Stresses

**Figure 3:**
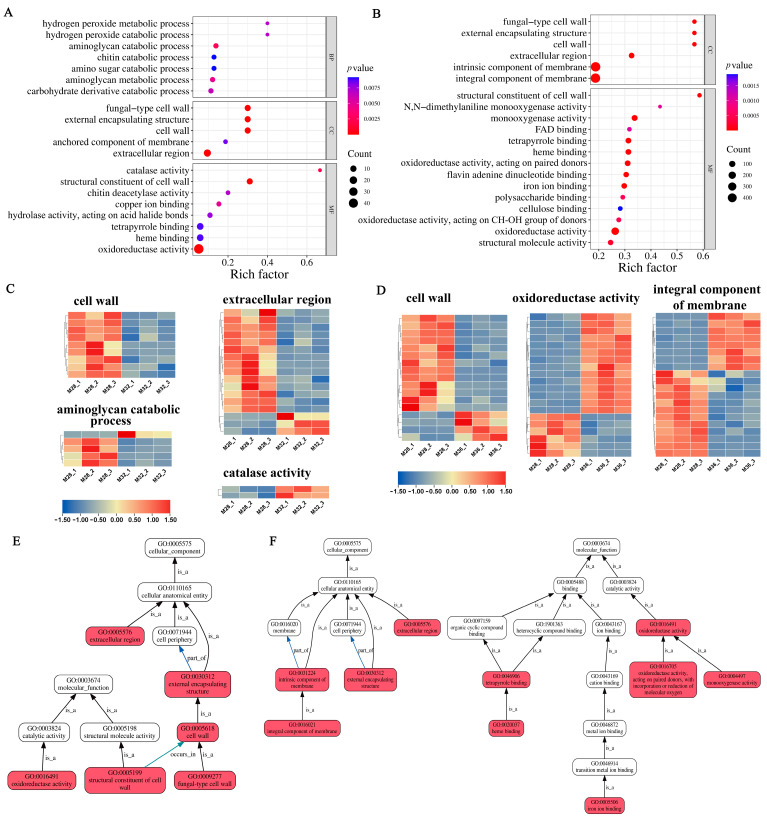
Functional enrichment analysis of DEGs under heat stress: (**A**) GO enrichment of DEGs under 32 °C heat stress. (**B**) GO enrichment of DEGs under 36 °C heat stress. (**C**) DEGs involved in critical pathways under heat stress at 32 °C. (**D**) DEGs involved in critical pathways under heat stress at 36 °C. (**E**) Directed acyclic graph of GO enrichment of DEGs under 32 °C heat stress. (**F**) Directed acyclic graph of the GO enrichment of DEGs under 36 °C heat stress.

To characterise the functions of the DEGs under mycelial heat stress, GO enrichment analysis was performed on the DEGs under heat stress at 32 °C and 36 °C. The top 20 GO terms (Figure 3A) enriched with DEGs under 32 °C heat stress included catalase activity, the cell wall, the aminoglycan catabolic process, and oxidoreductase activity. Under 32 °C stress, mycelia respond to environmental changes by regulating the production and clearance of ROS and regulating cell wall-related metabolic pathways. Therefore, DEGs involved in cellular constituents and oxidoreductase activity have been a focus of interest. In the cellular composition-related pathway, most DEGs associated with the structural constituents of the cell wall and extracellular region were expressed at significantly lower levels under 32 °C heat stress than under 28 °C heat stress (Figure 3C). Similarly, most DEGs involved in the aminoglycan catabolic process were significantly downregulated under heat stress at 32 °C. However, the expression of DEGs related to catalase activity was significantly greater at 32 °C than at 28 °C under heat stress (Figure 3C). Furthermore, DEGs under 32 °C heat stress are depicted in directed acyclic graphs in Figure 3E, which show the relationships of the GO terms. The results revealed that most of the highly enriched GO terms were classified as a cellular component.

The top 20 GO terms (Figure 3B) enriched with DEGs under 36 °C heat stress included the cell wall, integral components of the membrane, and oxidoreductase activity. The expression of most DEGs involved in the cell wall, an integral component of the membrane, was significantly lower under heat stress at 36 °C than at 28 °C (Figure 3D). Nevertheless, the expression of DEGs associated with oxidoreductase activity was significantly upregulated at 36 °C (Figure 3D). The GO terms enriched with DEGs under heat stress at 32 °C and 36 °C included the cell wall, oxidoreductase activity, and metal ion binding. These results suggest that genes related to cell wall composition, oxidoreductase activity, and metal ion binding may play important roles in the process of heat stress in mycelia. In addition, Figure 3F depicts DEGs under heat stress at 36 °C as a directed acyclic graph, and the results show that highly enriched GO terms were categorised into cellular component and molecular function.

### 3.5. Functional Enrichment Analysis of Unique DEGs Under Different Heat Stresses

**Figure 4:**
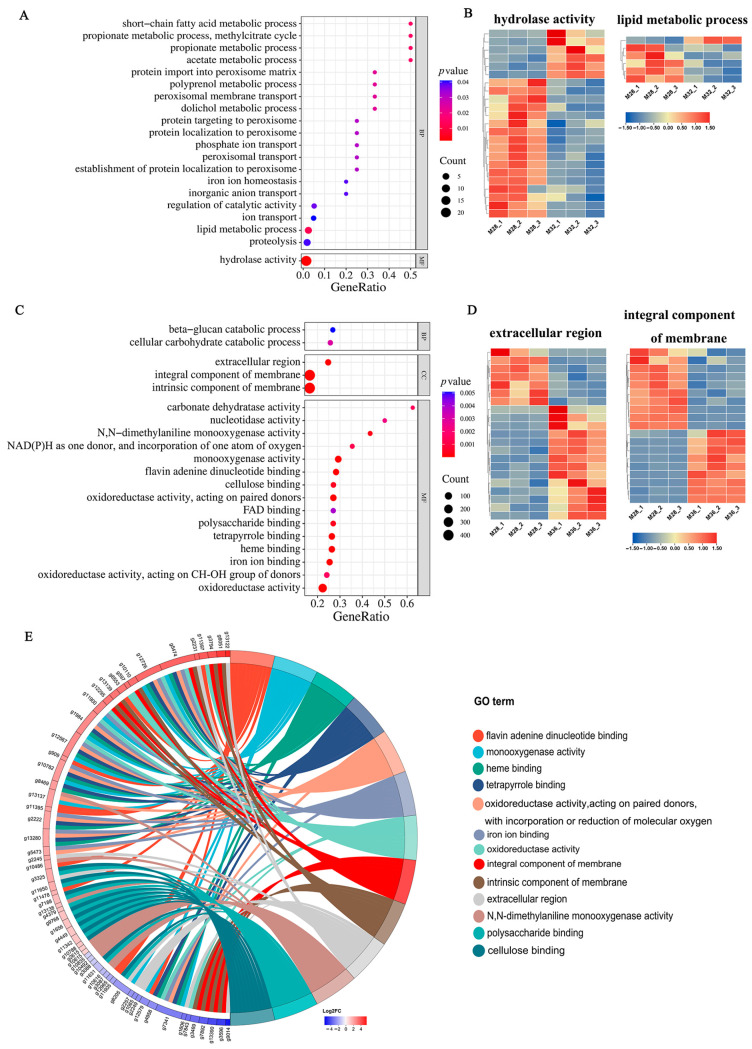
Functional enrichment analysis of specific DEGs under heat stress: (**A**) GO enrichment of specific DEGs under 32 °C heat stress. (**B**) Specific DEGs involved in the critical pathway under heat stress at 32 °C. (**C**) GO enrichment of specific DEGs under 36 °C heat stress. (**D**) Specific DEGs involved in critical pathways under heat stress at 36 °C. (**E**) GO enrichment analysis of DEGs in mycelia under 36 °C heat stress. Chords represent a detailed relationship between the expression levels of DEGs (left semicircle perimeter) and their enriched GO terms (right semicircle perimeter).

To study the differences in DEGs in mycelia under different heat stresses, GO enrichment analysis was performed on the unique DEGs under 32 °C and 36 °C heat stress. The top 20 GO terms enriched specifically by DEGs under 32 °C heat stress included hydrolase activity, lipid metabolic process, short-chain fatty acid metabolic process, proteolysis, and ion transport (Figure 4A). Most of the DEGs involved in hydrolase activity and lipid metabolism were significantly downregulated under heat stress at 32 °C (Figure 4B). The relevant genes in the hydrolase activity pathway under heat stress at 32 °C are shown in Table S2. The top 20 GO terms enriched specifically by DEGs under 36 °C heat stress included integral components of the membrane, extracellular region monooxygenase activity, and nucleotidase activity (Figure 4C). Further analysis revealed that under 36 °C heat stress, 9 DEGs were significantly upregulated and 10 DEGs were significantly downregulated among the specific DEGs involved in integral components of the membrane and intrinsic components of the membrane, indicating that these DEGs may be involved in the process of cell membrane damage caused by heat stress (Figure 4D). In addition, among the specific DEGs involved in the extracellular region, 7 DEGs were significantly downregulated and 14 DEGs were significantly upregulated. This may be the reason for the changes in the cell wall structure (Figure 4D). The enrichment chord plots for DEGs under heat stress at 36 °C revealed the most significant top GO terms (Figure 4E), among which flavin adenine dinucleotide binding, monooxygenase activity, heme binding, tetrapyrrole binding, and oxidoreductase activity were among the top five.

In summary, the DEGs unique to the 32 °C heat stress treatment were significantly enriched in biological processes, whereas most of the DEGs unique to the 36 °C heat stress treatment were enriched in cellular components. These findings indicate that mild heat stress at 32 °C affects mainly the biological processes of mycelia, whereas the structural components of cells are strongly affected under high-temperature stress at 36 °C.

### 3.6. Functional Enrichment Analysis of Coregulated DEGs Under Different Heat Stresses

**Figure 5:**
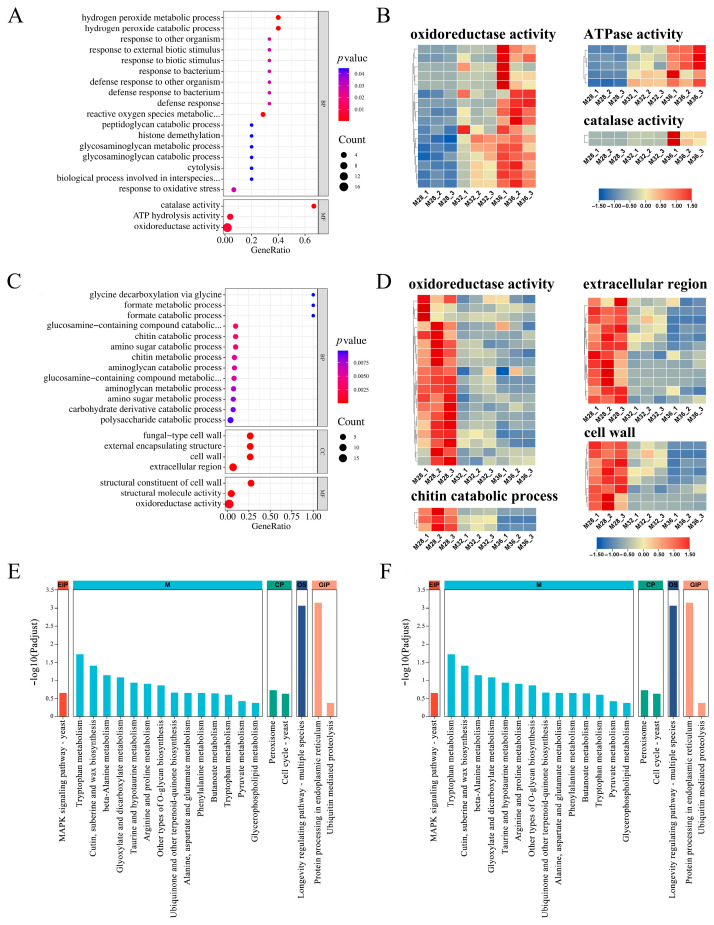
Functional enrichment analysis of common DEGs under different heat stresses.: (**A**,**B**) GO enrichment of common upregulated and common DEGs. (**C**,**D**) GO enrichment of common downregulated DEGs and common DEGs. (**E**,**F**) KEGG enrichment of common upregulated and common downregulated DEGs.

Under both 32 °C and 36 °C heat stress, 328 DEGs were coregulated, of which 158 DEGs were significantly upregulated and 170 DEGs were significantly downregulated. To further investigate the role of the coregulated DEGs under mycelial heat stress, GO enrichment and KEGG enrichment analyses were subsequently performed on the DEGs. Figure 5A shows the first 20 GO terms of 158 upregulated, coregulated DEGs, with 17 GO terms enriched in biological processes, including significant enrichment in the hydrogen peroxide catabolic process, hydrogen peroxide metabolic process, and ROS metabolic process. In terms of molecular function, significant enrichment was observed for oxidoreductase activity, ATP hydrolysis activity, and catalase activity, and in these three pathways, the number of DEGs gradually increased with increasing stress temperature (Figure 5B). Figure 5C shows that, under different types of heat stress, the most significantly enriched molecular functions of the co-downregulated DEGs were the external encapsulating structure, cell wall, fungal-type cell wall, extracellular region, structural constituent of the cell wall, structural molecule activity, and oxidoreductase activity. Under heat stress at 36 °C, the downregulation trend of DEGs was significantly more pronounced (Figure 5D). Genes that are usually upregulated or downregulated under heat stress are significantly enriched in oxidoreductase activity. It is speculated that genes related to hydrogen peroxide metabolism, oxidoreductase activity, ATP hydrolysis, and the cell wall may play important roles in heat stress processes.

In addition, KEGG analysis revealed that 158 co-upregulated DEGs under heat stress were significantly enriched in the longevity regulating pathway and protein processing in endothelial reticulum pathway, indicating that these two pathways are crucial for the mycelial response to heat stress at different temperatures. The significant enrichment of cutin, suberine, and wax biosynthesis may be related to the cell wall changes shown in Figure 1. In addition, the MAPK signaling pathway was also enriched, indicating the important role of signal transduction in the response of mycelia to heat stress (Figure 5E). Most of the co-downregulated DEGs were enriched in metabolic processes, including amino acid metabolism, sugar metabolism, and other pathways. In addition, the oxidative phosphorylation pathway was also significantly enriched (Figure 5F). These findings indicate that after being subjected to heat stress, multiple metabolic pathways of hyphae are inhibited, and the oxidative phosphorylation process is inhibited.

### 3.7. Genes Related to the Cell Wall Respond to Heat Stress

**Figure 6:**
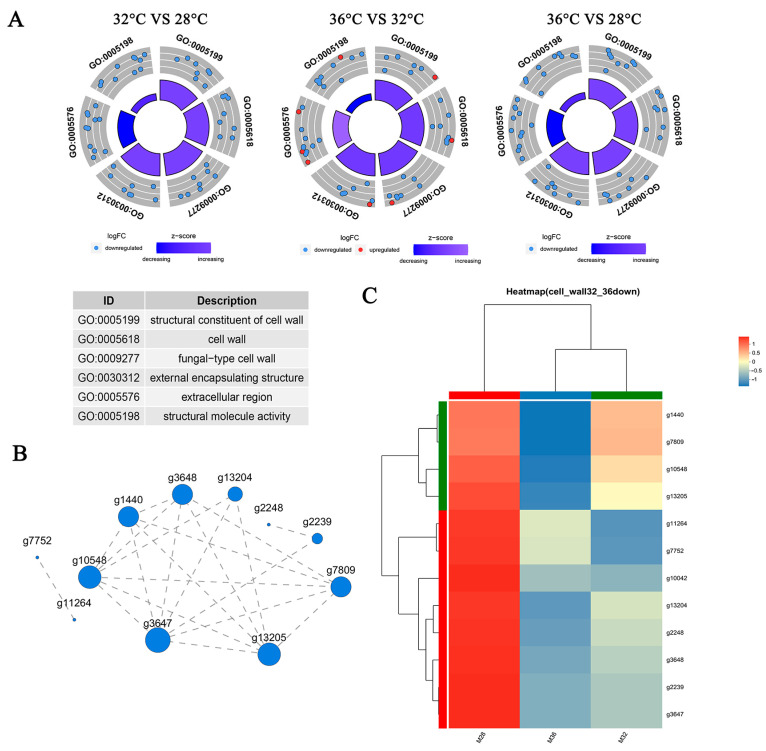
Correlation expression and heatmap of genes related to the cell wall response to heat stress: (**A**) Circle plot showing the DEGs and z-scores of “structural constituent of cell wall”, “cell wall”, “fungal-type cell wall”, “external encapsulating structure”, “extracellular region”, and “structural molecule activity” GO terms. The outer circle shows a scatter plot for each term of the fold change of the assigned genes. The inner circle shows the z-score of each GO term. The width of each bar corresponds to the number of genes within the GO term, and the color corresponds to the z-score. (**B**) Each node in the graph represents a gene, and the connections between nodes represent the correlation between gene expression. The larger the node is, the greater the number of expression correlations between the gene and other genes. (**C**) Each column represents a sample, and each row represents a gene. The colors in the graph represent the standardised expression levels of the genes in each sample, with red indicating relatively high expression levels in the sample and blue indicating relatively low expression levels.

The results of the physiological indicator detection described in Section 3.1 and the functional enrichment of the DEGs described in Section 3.4 revealed that cell walls may play a key role in heat stress in mycelia. To further analyse the molecular mechanism of the response of cell wall-related genes to heat stress, 12 cell wall-related DEGs were found to respond specifically to 36 °C heat stress. The results are shown in Table 2. The results revealed that 8 out of the 12 DEGs belong to the hydrophobic protein family, with two cerato-platanin-related secreted proteins, one laccase, and one glycoside hydrolase family 61 protein. Hydrophobic proteins are widely present in the cell wall of fungi, making the cell wall hydrophobic. Silencing hydrophobin reduces the resistance of *Ganoderma lucidum* mycelia to heat and salt stress [25]. Cerato platanin (CP) proteins are a class of secreted proteins produced in large quantities by filamentous fungi that participate in plant stress responses and can bind to fungal chitin oligomers [26]. Laccase participates in the formation of cell walls in higher plants and helps improve plant resistance to biotic and abiotic stress [27]. Glycoside hydrolases are present in almost all living organisms, are involved mainly in the breakdown and synthesis of sugars, and play important roles in cell wall metabolism, differentiation, and development. There was a total of five cell wall-related GO terms: “structural constituent of cell wall” (GO:0005199), “cell wall” (GO:0005618), “fungal-type cell wall” (GO:0009277), “external encapsulating structure” (GO:0030312), “extracellular region” (GO:0005576), and “structural molecule activity” (GO:0005198). Figure 6A shows the number and expression patterns of DEGs associated with these GO terms under heat stress at 32 °C and 36 °C. The results revealed that cell wall-related genes were downregulated under heat stress and that the number of DEGs increased with increasing heat stress temperature. In addition, eight DEGs were upregulated under 36 °C heat stress compared with those under 32 °C heat stress, indicating that there were differences in the expression patterns of DEGs under heat stress at different temperatures. Figure 6B shows the expression correlation of 12 DEGs, most of which were closely related. In addition, heatmaps were used to analyse the expression patterns of the 12 DEGs after heat stress. As shown in Figure 6C, the expression levels of 12 DEGs were significantly downregulated after heat stress. Among them, nine DEGs presented the same trend; that is, the higher the temperature was under heat stress, the lower the gene expression level was.

### 3.8. qRT–PCR Validation of the RNA-Seq Data

**Figure 7:**
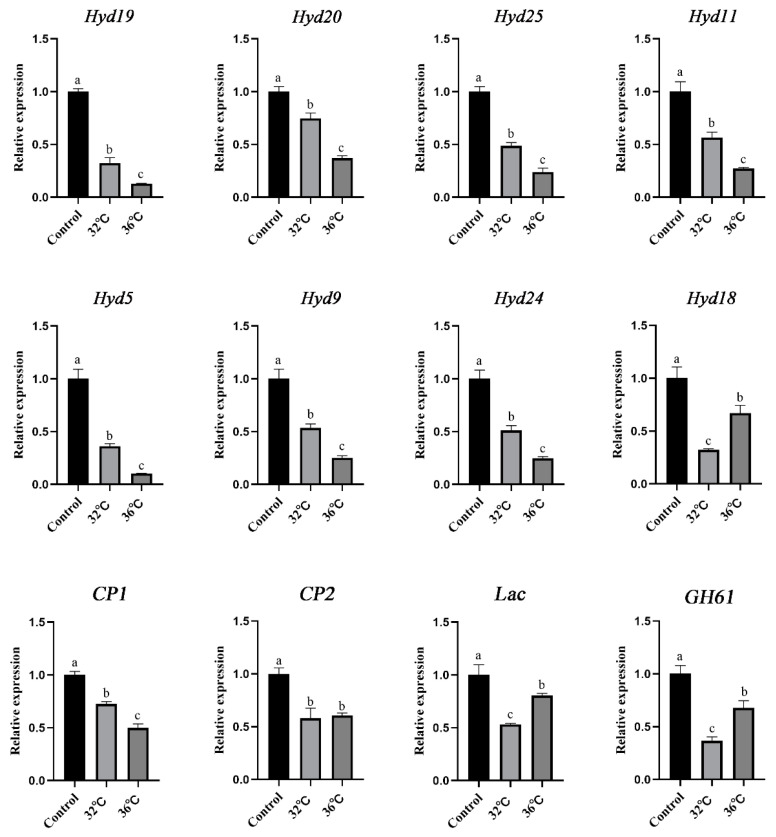
Expression analysis of genes related to the cell wall in response to heat stress. Different letters indicate significant differences for the comparison of samples (*p* < 0.05 according to Duncan’s test).

To further validate the reliability of the transcriptome data, the expression of 12 DEGs related to the cell wall response to heat stress was detected. As shown in Figure 7, the expression levels of 12 DEGs in mycelia decreased after being subjected to heat stress. Among the eight *hyd* genes, seven presented the same trend, and their expression levels gradually decreased with increasing stress temperature. Only the expression level of *hyd18* was slightly greater under 36 °C stress than under 32 °C stress. The *Lac* gene and *GH61* presented the same expression pattern, with a decrease in expression levels after heat stress; however, the expression level under heat stress at 36 °C was greater than that at 32 °C. Research has shown that the CP protein is found only in fungi and mainly acts on the cell wall, possibly participating in cell wall remodelling and enlargement during fungal cell growth and spore formation. In this study, the gene expression levels of *CP1* and *CP2* were significantly downregulated after heat stress, and their expression patterns were consistent with those of *hyd* genes. Overall, the expression patterns observed in the RNA-seq data were consistent with those analysed via qRT–PCR.

## 4. Discussion

The high-temperature environment is one of the main factors affecting the yield and quality of shiitake mushrooms in China. In this study, the colony diameter of *P. ostreatus* mycelia significantly decreased under heat stress at 32 °C and 36 °C, with growth inhibition rates of 19.07% and 84.13%, respectively. *Ganoderma lucidum* and *Schizophyllum commune* can also experience a decrease in the mycelial growth rate under heat stress [28,29]. Our research results are similar to those of previous researchers. The cell wall is the first line of defense for organisms in response to stress. When fungal cells are subjected to environmental stress, they initiate a series of metabolic pathways to maintain cell shape and cell wall integrity (CWI) [30]. Environmental conditions of stress may lead to changes in the composition of the yeast cell wall, disorganisation of its assembly, and disruption of its structure [31]. In addition, high temperature triggers metabolic disorders in *Pleurotus tuber-regium*, and many hydrolytic enzymes are released to promote the degradation of the cell wall and cytoplasm; at this time, many β-1,3-glucanosyltransferase and microtubule proteins are synthesised, which participate in the renewal and repair of the cell wall to maintain its normal morphology and function [32]. This study revealed that the cell wall of mycelia became thicker and that the density decreased after heat stress. Moreover, the activities of chitinase and β-1,3-glucanase were significantly reduced. Previous studies have shown that high-temperature treatment of mushroom mycelia can lead to an uneven distribution of chitin in the cell wall, reduce the content of mannose in the cell wall, and increase the relative porosity of the cell wall [3]. The same situation occurs in plants: high temperatures can reduce the sugar content of cellulose and hemicellulose in *Zea mays* L. [33], and heat stress can lead to a decrease in the polymer content and structural changes in the cell wall of coffee leaves. Heat stress can lead to a decrease in the cell wall polymer content in *Coffea arabica* L. and changes in the structure of coffee leaf cell walls [34]. This finding is consistent with our findings that the appearance and composition of mycelial cell walls change after heat stress. These results indicate that the mycelial cell wall is severely affected after heat stress.

High temperature is one of the main environmental factors affecting the growth and development of large fungi [35]. Heat stress is a common environmental stress that has a wide range of effects on the physiology and metabolism of organisms [36,37]. This study revealed that the number of DEGs under 36 °C heat stress was 4.62-fold greater than that under 32 °C heat stress. The higher the stress temperature is, the more DEGs there are, and the stronger the stress response within the mycelia is. These findings indicate that there are significant differences in transcription levels in cells under different temperature stresses. Further research revealed that, under 32 °C stress, DEGs were significantly enriched in biological processes such as hydrolytic enzyme activity, lipid metabolism, short-chain fatty acid metabolism, protein hydrolysis, and ion transport. When the temperature increased to 36 °C, the cellular structural components, including the composition of the membrane, intrinsic components, and extracellular regions, were significantly affected. The cell wall and membrane can be considered the first line of defense for cells to resist external pressure. Previous studies have shown that, under mild heat stress, the integrity of the mycelial cell membrane is better; under extreme heat stress, the integrity of the mycelial cell membrane is severely damaged [38]. Heat stress causes damage to the mycelial cell membrane of *Ganoderma lucidum* and increases fluidity and permeability; moreover, disruption of the cell membrane results in the loss of cytoplasmic contents and even cell death [39]. The high temperature disrupted the membrane structure of the mycelium of *Pleurotus tuber-regium*, protein hydrolysing enzymes were released, and the change in osmotic pressure destroyed the structure of the organelles, leading to a loss of balance in the synthesis and hydrolysis of the cell contents [32]. Previous studies have shown that the disruption of the chitin synthase gene leads to sparser hyphae, rougher surfaces, and shorter aerial hyphae. They also lead to increased sensitivity to the cell wall and membrane stress, resulting in thinning of the cell wall [40]. Our results are consistent with previous phenotype results, indicating that the cell membrane and cell wall play important roles in stress.

In recent years, the analysis of heat stress response mechanisms has become a hot topic in the field of edible fungi. Research has shown that the increase in ROS under heat stress is the key factor causing cell damage [41]. Therefore, many studies have investigated gene functions, transcription factors, and their regulatory mechanisms centered around ROS. For example, in *P. ostreatus*, exogenous salicylic acid treatment reduces intracellular ROS levels in shiitake mushrooms under heat stress but increases cytoplasmic Ca^2+^ levels [8]. In addition, nitric oxide induces the expression of alternative oxidase genes to regulate ROS and increase the tolerance of mycelia to heat stress [42]. In *Pleurotus eryngii*, exogenous CaCl_2_ transmits Ca^2+^ signals through *PeCaMK1* to increase antioxidant activity, reduce ROS accumulation, and ultimately delay quality deterioration [43]. In this study, we identified 158 upregulated DEGs regulated by both 32 °C and 36 °C, which were significantly enriched in the hydrogen peroxide catabolic process, the hydrogen peroxide metabolic process, and the ROS metabolic process, providing candidate gene libraries for further gene function research. In addition, 170 co-downregulated DEGs were further identified under 32 °C and 36 °C heat stress, and these genes were significantly enriched in cell wall-related pathways, such as glucamine-containing compound catabolic and metabolic processes and chitin catabolic and metabolic pathways. Interestingly, glucosamine-containing compounds are components of chitin, glycoproteins, and proteoglycans. A similar situation has been observed in other edible mushrooms. Heat stress results in the severe disruption of *Ganoderma lingzhi* cell wall integrity, and proteomic analyses revealed that proteins related to cell wall macromolecule metabolism were downregulated [44]. In addition, edible mushrooms have other response pathways under heat stress, and transcriptomic analyses revealed that *Agaricus bisporus* responds to heat stress by initiating the expression of antioxidant enzymes and heat shock protein genes [45]. The proteome and transcriptome revealed the effects of heat shock proteins and indoleacetic acid metabolic processes on heat tolerance in *Lentinula edodes* [46]. Previous studies have reported significant changes in the transcription levels of cell wall-related genes during the plant heat stress response. For example, when *Brassica rapa* L. is exposed to high-temperature conditions, the expression of cell wall enzymes or proteins such as arabinogalactan protein, β-glucosidase, cellulose synthase, expansin, extensin, glycosyl transferase, pectin esterase, and xylosidase is upregulated two to three-fold [47]. When *Brassica napus* is heated, the expression of the extended protein-encoding gene (*EXP*5) and pectin methylesterase (*PME*) gene decreases nearly tenfold [48]. Similar results were observed in *Populus euphratica*, with 13 genes encoding *EXP* downregulated [49]. These results indicate that plant cell-wall-related genes play important roles in the heat stress response. In fungi, previous studies have shown that the cell wall plays an important role in mycelial morphogenesis, mechanical strength, and interactions with surrounding environments such as soil, plants, and pathogens [50]. Our research is similar to previously reported results in plants, not only elucidating the important role of cell wall-related metabolism in the heat stress response of edible mushroom mycelia but also providing a candidate gene library for further in-depth research.

## 5. Conclusions

In summary, under heat stress at 32 °C and 36 °C, the growth rate of the mycelia of *P. ostreatus* significantly decreased, with growth inhibition rates of 19.07% and 84.13%, respectively. The transmission electron microscopy results revealed that the cell wall and cell membrane morphology of the mycelium changed under heat stress. Through transcriptome data analysis, DEGs were significantly enriched in various biological processes under 32 °C stress. When the temperature increased to 36 °C, the cellular structural components, including the membrane composition, intrinsic components, and extrinsic components, were significantly affected. Second, 12 DEGs related to cell walls that can respond specifically to extreme heat stress response processes were identified, providing a candidate gene library for further in-depth research.

## Figures and Tables

**Table 1:** Summary of the trimming and reading localisation results of the sequences generated by the growth of *P. ostreatus* mycelium at 28 °C and heat stress at 32 °C and 36 °C, respectively.

Sample	Raw Reads	Clean Reads	Total Mapped	Multiple Mapped	Uniquely Mapped
M28_1	48052110	47638650	37745127 (79.23%)	326372 (0.69%)	37418755 (78.55%)
M28_2	44714218	44328286	35661000 (80.45%)	297190 (0.67%)	35363810 (79.78%)
M28_3	43469900	43070352	34767913 (80.72%)	276462 (0.64%)	34491451 (80.08%)
M32_1	48827640	48430718	38597088 (79.70%)	322669 (0.67%)	38274419 (79.03%)
M32_2	52328856	51949474	41105356 (79.13%)	349492 (0.67%)	40755864 (78.45%)
M32_3	54948006	54518710	43782677 (80.31%)	355754 (0.65%)	43426923 (79.66%)
M36_1	79999062	79401364	62828943 (79.13%)	533955 (0.67%)	62294988 (78.46%)
M36_2	47631544	47215264	37457481 (79.33%)	320792 (0.68%)	37136689 (78.65%)
M36_3	43746956	43421102	33967284 (78.23%)	293893 (0.68%)	33673391 (77.55%)

**Table 2:** Functions of the 12 DEGs associated with the cell wall response to heat stress.

Gene ID	Name	Gene Function
g1440	*Hyd5*	hydrophobin modified 4
g7809	*Hyd11*	putative class I hydrophobin superfamily
g10548	*Hyd25*	class I hydrophobin superfamily
g13205	*Hyd19*	hydrophobin
g11264	*GH61*	glycoside hydrolase family 61 protein
g7752	*Hyd18*	fungal hydrophobin
g10042	*Lac*	laccase
g13204	*Hyd20*	class I hydrophobin superfamily
g2248	*CP1*	cerato-platanin-related secreted protein1
g3648	*Hyd9*	class I hydrophobin superfamily
g2239	*CP2*	cerato-platanin-related secreted protein2
g3647	*Hyd24*	class I hydrophobin superfamily

## Data Availability

All data generated or analysed during this study are included in this published article.

## Supplementary Materials

The following supporting information can be downloaded at: https://www.mdpi.com/article/10.3390/jof11040266/s1. Table S1: Primers used in this study; Table S2: Gene annotation of GO pathway (hydrolase activity) under 32 °C heat stress.
