# Supplementary material for: Transcriptome Analysis Revealed That Cell Wall Regulatory Pathways Are Involved in the Tolerance of Pleurotus ostreatus Mycelia to Different Heat Stresses
Source: J Fungi (Basel). 2025 Mar 30;11(4):266. doi: 10.3390/jof11040266 (PMC12028245; doi:10.3390/jof11040266)
Supplement: Supplementary file 1 [file jof-11-00266-s001.zip › jof-3513365-supplementary.pdf]

Table S1. Primers used in this study.

Primer	Sequence (5′→3′)
qPCR_*Hyd5*-F	TGGATATCAAAGGCGTTGTAGG
qPCR_*Hyd5*-R	GCAGCCAATGTTGACAAGG
qPCR_*Hyd9*-F	GTAGCTGTAACGCACAAACC
qPCR_*Hyd9*-R	GGCAACACAACCGACATTG
qPCR_*Hyd11*-F	CGAGTTCAGCTTGCAATGC
qPCR_*Hyd11*-R	GGAGTGCAGCCAATGTTAAC
qPCR_*Hyd18*-F	TGGGTTGGAGCACTTTGG
qPCR_*Hyd18*-R	GAAGTGGACATTTTCGCAGC
qPCR_*Hyd19*-F	ATTGTGCTCGGTCCCATC
qPCR_*Hyd19*-R	AGCACCCAACGACAATGAG
qPCR_*Hyd20*-F	AACAGTCGTTGGCCTAGC
qPCR_*Hyd20*-R	CTTTCAATGTGAGATCCTTGCAG
qPCR_*Hyd24*-F	GTAGCTGTAACGCACAAACC
qPCR_*Hyd24*-R	GGCAACACAACCGACATTG
qPCR_*Hyd25*-F	TCGGAAACATCTATGCGTCTC
qPCR_*Hyd25*-R	GGTGCATCCAACATTGACAAG
qPCR_*GH61*-F	CCCGGTGCTCAGTTCTATATG
qPCR_*GH61*-R	GTGTAGTCTGTGATGCCGTAG
qPCR_*Lac*-F	ACAATGAAGACCAGTACCAGC
qPCR_*Lac*-R	AGAATGTCCGGGAACAATGG
qPCR_*CP1*-F	GTCGTTCAATGGTACAATGG
qPCR_*CP1*-R	ACAAGCCACAAGCAGATG
qPCR_*CP2*-F	AGCATACGACGATCATAGC
qPCR_*CP2*-R	CAGCAGGATTGGTATAGGTT
qPCR_*tub*-F	AGGCTTTCTTGCATTGGTACACGC
qPCR_*tub*-R	TATTCGCCTTCTTCCTCATCGGCA

Table S2. Gene annotation of GO pathway (hydrolase activity) under 32°C heat stress.

Gene ID	Gene Description
g1936	subtilisin-like protein
g801	carbohydrate esterase family 4 protein
g10151	agmatinase
g11048	protein-tyrosine phosphatase
g819	glycoside hydrolase family 30 protein
g7346	subtilisin-like protein
g9093	cyclin-dependent protein kinase inhibitor
g6914	acid protease
g6586	p-loop containing nucleoside triphosphate hydrolase protein
g2236	-
g2799	-
g10707	proline-specific peptidase
g8728	amidohydrolase 2
g6894	acid protease
g1126	glycoside hydrolase family 16 protein
g8221	glycoside hydrolase family 23 protein
g4418	rad26-like snf2 family dna-dependent atpase
g3306	protein
g13181	acid protease
g10500	acetyl- hydrolase
g6777	carbohydrate esterase family 16 protein
g10678	extracellular guanyl-specific ribonuclease
g7546	glycoside hydrolase family 79 protein
